# There is no association between autosomal dominant polycystic kidney disease and left ventricular non-compaction cardiomyopathy: a cardiac magnetic resonance imaging study

**DOI:** 10.1186/1532-429X-18-S1-Q47

**Published:** 2016-01-27

**Authors:** Shadi Akhtari, Shingo Kato, James D Chang, Theodore I Steinman, Warren J Manning

**Affiliations:** grid.239395.70000000090118547Cardiology, Beth Israel Deaconess Medical Center, Boston, MA USA

## Background

Autosomal dominant polycystic kidney disease (ADPKD) is the most common familial renal disorder. Left ventricular non-compaction cardiomyopathy (LVNC) is a rare cardiac condition resulting primarily from interruption of the normal embryonic process of myocardial compaction. Genes responsible for ADPKD have been linked to disorganized myocardial arrangement in animal models. In addition, there are multiple published case reports of ADPKD and LVNC occurring in the same patient. Therefore, we sought to examine possible association of ADPKD with LVNC in a large cohort of patients using cardiac magnetic resonance (CMR) imaging.

## Methods

In this descriptive, retrospective study, we analyzed 126 CMR scans from 36 patients with known ADPKD, who had undergone serial CMR testing at Beth Israel Deaconess Medical Center, Boston, MA as part of HALT-PKD trial. We examined for both the Peterson's CMR criteria (non-compacted/compacted ratio > 2.3 in diastole on long-axis images) and Jenni's echocardiographic criteria (non-compacted/compacted ratio > 2.0 at end-systole on short-axis images) in order to identify potential cases of LVNC. A random sample of 10 scans was independently analyzed by a second reader to assess for inter-observer reproducibility.

## Results

None of the patients (0/126 scans) met the diagnostic criteria for LVNC. There was 100% inter-observer agreement between the two readers in the 10 random scans analyzed.

## Conclusions

In this study of 36 patients with ADPKD, we found no association between ADPKD and LVNC using cardiac magnetic resonance imaging. These data suggest that the case reports in the literature likely represent spurious/random incidents of the coexistence of both diseases rather than a true association.

